# Fertility Preservation in Childhood Cancer: Endocrine Activity in Prepubertal Human Testis Xenografts Exposed to a Pubertal Hormone Environment

**DOI:** 10.3390/cancers12102830

**Published:** 2020-09-30

**Authors:** Marsida Hutka, Prashant Kadam, Dorien Van Saen, Natalie Z. M. Homer, Jaime Onofre, W. Hamish B. Wallace, Lee B. Smith, Jan-Bernd Stukenborg, Ellen Goossens, Rod T. Mitchell

**Affiliations:** 1 Medical Research Council (MRC) Centre for Reproductive Health, The University of Edinburgh, The Queen’s Medical Research Institute, 47 Little France Crescent, Edinburgh EH16 4TJ, UK; marsida.hutka@gmail.com (M.H.); lee.smith@ed.ac.uk (L.B.S.); 2 Biology of the Testis, Research Laboratory for Reproduction, Genetics and Regenerative Medicine, Vrije Universiteit Brussel (VUB), Laarbeeklaan 103, 1090 Brussels, Belgium; prashant.k.vet@gmail.com (P.K.); Dorien.Van.Saen@vub.be (D.V.S.); jaimeonofrem@gmail.com (J.O.); Ellen.Goossens@vub.be (E.G.); 3 Edinburgh CRF Mass Spectrometry Core, Centre for Cardiovascular Science, The University of Edinburgh, The Queen’s Medical Research Institute, 47 Little France Crescent, Edinburgh EH16 4TJ, UK; n.z.m.homer@ed.ac.uk; 4 Department of Oncology and Haematology, Royal Hospital for Sick Children, 9 Sciennes Road, Edinburgh EH9 1LF, UK; hamish.wallace@nhs.net; 5 School of Environmental and Life Sciences, University of Newcastle, Callaghan, 2308 New South Wales, Australia; 6 NORDFERTIL Research Lab Stockholm, Childhood Cancer Research Unit, Department of Women’s and Children’s Health, Karolinska Institutet, and Karolinska University Hospital, Visionsgatan 4, 171 64 Solna, Sweden; jan-bernd.stukenborg@ki.se; 7 Department of Diabetes and Endocrinology, Royal Hospital for Sick Children, 9 Sciennes Road, Edinburgh EH9 1LF, UK

**Keywords:** prepubertal human testis, childhood cancer, gonadotoxicity, side effects, steroidogenesis, testosterone, hCG, FSH, fertility preservation, xenotransplantation

## Abstract

**Simple Summary:**

Substantial strides have been made in treating childhood cancers; however, as a result of chemotherapy and radiotherapy, young males experience long-term side effects, including impaired fertility. Whilst prepubertal testicular tissue can be cryopreserved prior to gonadotoxic treatments, it remains to be determined how to generate mature gametes from the immature human testis tissue. Development of immature germ cells into sperm is a complex process, which is supported by mature Sertoli cells and testosterone produced from Leydig cells. We used an established testicular xenotransplantation model to investigate the effect of puberty hormones, known as gonadotrophins, on functional maturation of the spermatogonial stem cell (SSC) niche. Limited testosterone production and partial maturation of Sertoli cells occurred in prepubertal testis grafts, suggesting that longer periods of grafting and/or identification of additional factors are required to develop testicular transplantation as a model for fertility preservation in male survivors of childhood cancer.

**Abstract:**

Survivors of childhood cancer are at risk for long-term treatment-induced health sequelae, including gonadotoxicity and iatrogenic infertility. At present, for prepubertal boys there are no viable clinical options to preserve future reproductive potential. We investigated the effect of a pubertal induction regimen with gonadotrophins on prepubertal human testis xenograft development. Human testis tissue was obtained from patients with cancer and non-malignant haematological disorders (*n* = 6; aged 1–14 years) who underwent testis tissue cryopreservation for fertility preservation. Fresh and frozen-thawed testis fragments were transplanted subcutaneously or intratesticularly into immunocompromised mice. Graft-bearing mice received injections of vehicle or exogenous gonadotrophins, human chorionic gonadotrophin (hCG, 20 IU), and follicle-stimulating hormone (FSH, 12.5 IU) three times a week for 12 weeks. The gross morphology of vehicle and gonadotrophin-exposed grafts was similar for both transplantation sites. Exposure of prepubertal human testis tissue xenografts to exogenous gonadotrophins resulted in limited endocrine function of grafts, as demonstrated by the occasional expression of the steroidogenic cholesterol side-chain cleavage enzyme (CYP11A1). Plasma testosterone concentrations (0.13 vs. 0.25 ng/mL; *p* = 0.594) and seminal vesicle weights (10.02 vs. 13.93 mg; *p* = 0.431) in gonadotrophin-exposed recipient mice were comparable to vehicle-exposed controls. Regardless of the transplantation site and treatment, initiation and maintenance of androgen receptor (AR) expression were observed in Sertoli cells, indicating commitment towards a more differentiated status. However, neither exogenous gonadotrophins (in castrated host mice) nor endogenous testosterone (in intact host mice) were sufficient to repress the expression of markers associated with immature Sertoli cells, such as anti-Müllerian hormone (AMH) and Ki67, or to induce the redistribution of junctional proteins (connexin 43, CX43; claudin 11, CLDN11) to areas adjacent to the basement membrane. Spermatogonia did not progress developmentally but remained the most advanced germ cell type in testis xenografts. Overall, these findings demonstrate that exogenous gonadotrophins promote partial activation and maturation of the somatic environment in prepubertal testis xenografts. However, alternative hormone regimens or additional factors for pubertal induction are required to complete the functional maturation of the spermatogonial stem cell (SSC) niche.

## 1. Introduction

Improved survival rates for childhood cancers have resulted in an increasing awareness of long-term treatment-related toxicities, affecting reproductive and endocrine function [1]. Unlike adult men with cancer, sperm cryobanking prior to initiating life-saving therapies is not feasible for prepubertal boys who are not yet producing mature germ cells, posing a challenge for fertility preservation in this cohort of patients [2,3,4,5,6,7,8]. At present, prepubertal boys do not have clinical options that protect and preserve their future fertility. A potential approach to address this issue is to cryopreserve testicular tissues that contain SSCs prior to initiating any gonadotoxic cancer therapy [6]. Fayomi and colleagues have demonstrated that the autologous transplantation of cryopreserved prepubertal non-human primate testis tissue can produce functional sperm that can subsequently be used to generate live offspring through intracytoplasmic sperm injection [9]. Although this study is particularly encouraging and holds promise for young boys, applications using cryopreserved prepubertal human testis tissue remain experimental. 

Germ cell differentiation into sperm is an extremely well-orchestrated process in which the two major testicular somatic cell populations in the SSC niche—i.e., Sertoli and Leydig cells—act as central regulators. In prepubertal human testis, immature Leydig cells and spindle-shaped fibroblast-like cells (adult Leydig cell progenitors) reside in the interstitial compartment [10], whereas immature Sertoli cells are dispersed throughout the seminiferous cords and intermingled with undifferentiated germ cells. 

Luteinizing hormone (LH) and FSH, referred to as gonadotrophins, are the pivotal endocrine hormones required for the development of the immature testis [11]. Gonadotrophins and testosterone levels fall after mini-puberty (~ 6 months after birth) and reach the prepubertal nadir, whereas the onset of puberty is heralded by the secretion of gonadotrophins and the resumption of testosterone production. FSH, acting through the FSH receptor (FSHR) on the surface of Sertoli cells, regulates the number of Sertoli cells before puberty, which in turn determines the spermatogenic output [12]. LH binds to the LH receptor (LHR) on Leydig cells and stimulates testicular steroidogenesis. In the postnatal testis, Leydig cell progenitors express a truncated form of the LHR mRNA, whereas immature and adult-type Leydig cells express the full-length transcript [13]. As puberty approaches, Leydig and Sertoli cells undergo maturation and transduce signals from LH and FSH into the paracrine regulation of SSC development into sperm [14].

Testis tissue xenotransplantation has proven to be a valuable experimental platform for investigating testicular development and its endocrine function in many species, including non-human primates [15]. Several groups have attempted to xenotransplant fresh/cryopreserved prepubertal human testis pieces at different sites. Long-term germ cell survival and differentiation up to the stage of primary spermatocytes was reported when prepubertal human testis tissue was grafted at various sites, including subcutaneous [16], scrotal [17], and intratesticular [18,19] locations. To stimulate further germ cell maturation, Van Saen and colleagues exposed pre(peri)pubertal human testis grafts to exogenous FSH; however, this did not improve germ cell survival and differentiation [18]. Providing a suitable hormonal environment is critical for achieving successful testicular development. The correlation between the rise of the gonadotrophins concentration and the onset of puberty is regarded as a causal relationship. However, a study directly measuring the impact of combined gonadotrophins, as occurs naturally in pubertal onset in humans, on prepubertal human testis graft development has not been performed. To this end, we investigated the effect of exogenous administration of a combination regimen of gonadotrophins, hCG (i.e., LH analogue), and FSH on the steroidogenic potential of testicular interstitial cells and the maturation of the Sertoli cell population in prepubertal human testis grafts. In addition, we sought to assess the impact of the transplantation site on testicular graft integrity and germ cell development.

## 2. Results

Pre(peri)pubertal human testicular tissues were obtained from patients (*n* = 6; aged 1–14 years) with cancer and non-malignant haematological disorders who underwent testis tissue cryopreservation for fertility preservation. Patient details are shown in Table 1.

Cryopreservation was performed using an uncontrolled slow freezing protocol, as previously described [18,20]. Cryovials containing frozen human pre(peri)pubertal testis pieces were removed from liquid nitrogen and immediately placed into a 37 °C water bath. The cryoprotectant was then removed by washing the testis pieces twice in Dulbecco’s modified Eagle’s medium (DMEM; Invitrogen, Merelbeke, Belgium) supplemented with 10% Human Serum Albumin (HSA; Vitrolife, Gothenburg, Sweden) [21]. The pieces were kept on ice until grafting. Castrated mice (*n* = 21) were grafted subcutaneously. Intact mice (*n* = 12) were grafted simultaneously under the dorsal skin and intratesticularly. Testis tissue was transplanted intratesticularly in the remaining four intact mice.

The experimental endpoints evaluated included histological and immunofluorescent/immunohistochemical analyses of xenografts. The seminal vesicle weight was also recorded as a reliable biomarker of circulating testosterone concentration. Testosterone levels produced by prepubertal human testis grafts were determined by liquid chromatography-tandem mass spectrometry (LC-MS/MS).

### 2.1. Graft Recovery Rate and Graft Weight

Overall, the graft recovery rates for subcutaneous and intratesticular transplants were 39% and 65%, respectively. Grafts were recovered from all the prepubertal patients; however, none of the intratesticular grafts from the peripubertal 13-year-old boy with spermatocytes in his testes before grafting could be retrieved from the host mice. An overview of the graft recovery rate can be found in Table 2.

**Figure 1:**
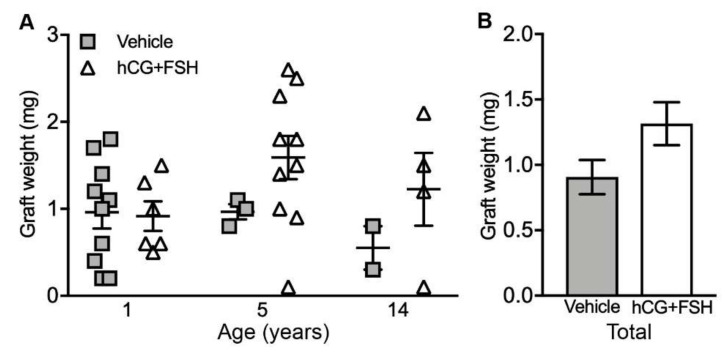
Graft weights from the castrate recipient mice exposed to either vehicle or gonadotrophins (hCG + FSH). (**A**) Individual data points represent the weight of a single subcutaneous xenograft, *n* = 15–20 grafts per group; *p =* 0.113. (**B**) Graph shows overall mean data. Statistical significance was determined using two-way ANOVA. Data are presented as mean ± SEM.

For the fresh testis tissue transplanted subcutaneously into castrate mice, the graft weights did not differ significantly between vehicle- and gonadotrophin-exposed groups (0.91 vs. 1.32 mg; *p* = 0.113; Figure 1a,b).

### 2.2. Histological Evaluation and Localisation of Prepubertal Human Testis Grafts

**Figure 2:**
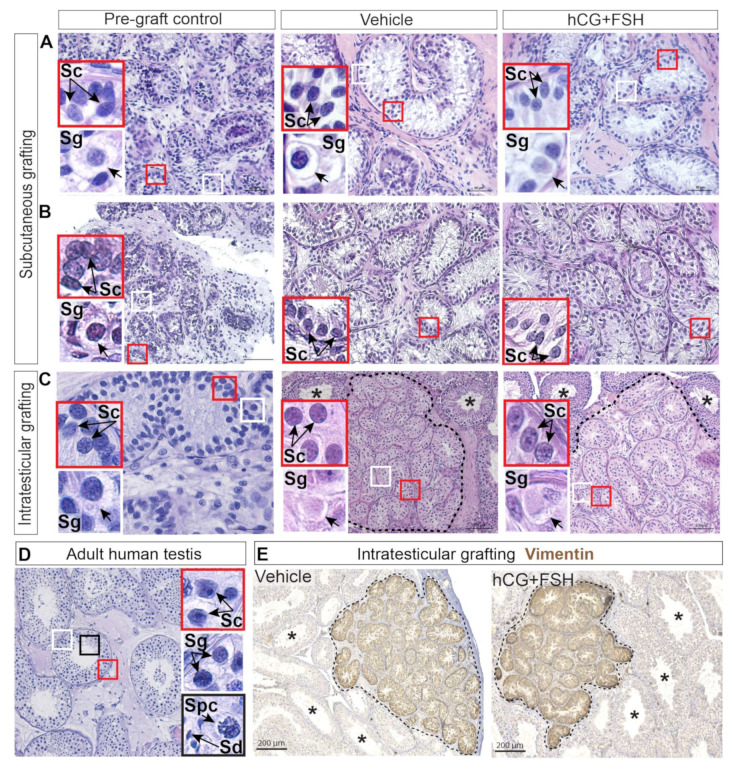
Haematoxylin and eosin staining and localisation of intratesticular prepubertal human testis grafts. (**A**–**C**) Histological examination of pre-graft controls (**A**. 1-year-old, **B**. 5-year-old, and **C**. 14-year-old) and grafts revealed normal appearance of the interstitium and seminiferous cords. (**D**) Adult human testis. (**E**) Expression of Vimentin denotes vehicle and hCG+FSH-treated prepubertal human testis tissue transplanted intratesticularly. Dashed lines depict the outline of grafts transplanted into the mouse testis. Host mouse seminiferous tubules with complete spermatogenesis denoted by an asterisk (*). Insets show higher magnification of Sertoli and germ cells. Sc: Sertoli cell. Sg: spermatogonium. Spc: spermatocyte. Sd: spermatid. (**A**–**C**) Scale bars: 50 μm, 100 μm. (**D**) Scale bar: 100 μm. (**E**) Scale bars: 200 μm.

Histological analysis of the xenografts showed preservation of the testis tissue architecture and morphology, with well-defined seminiferous cords and interstitial compartments across all the experimental groups (Figure 2a–c). Spermatogonia are the most advanced germ cell type in prepubertal human testis [22,23,24]. At puberty, spermatogonia differentiate into spermatocytes, the latter give rise to spermatids; and subsequently, spermatids develop into spermatozoa [25]. Spermatocytes were found prior to xenografting in the testis of a 13-year-old peripubertal boy (Figure S1), whereas none of the five prepubertal patients had spermatocytes in their testes before xenotransplantation. Previous studies showed the same prepubertal pattern, with seminiferous cords containing spermatogonia as the most advanced germ cell type in pre-graft controls [17,18,19,26]. Progression through meiosis did not occur in prepubertal testis grafts from either treatment group when compared to adult human testis tissue (Figure 2a–d). Immature Sertoli cells exhibiting elongated-to-oval nuclei with regular outlines [27] were found in pre-graft controls and grafts (Figure 2a–c), whereas mature Sertoli cells with an irregular nuclear shape and a prominent nucleolus [28] were identified in adult testis tissue (Figure 2d).

Intratesticular xenografts could be easily identified and distinguished from the surrounding mouse testicular tissue by the expression of Vimentin, a class III intermediate filament protein that is found in Sertoli cells from foetal life onwards (Figure 2e).

### 2.3. Effect of Exogenous Gonadotrophins on Steroidogenesis in Prepubertal Human Testis Grafts

In the present study, the effect of exogenous gonadotrophins on the endocrine activity of the prepubertal interstitial compartment was assessed by analysing three parameters: (i) expression of the steroidogenic enzyme, CYP11A1; (ii) recipient mouse plasma testosterone concentrations; and (iii) recipient mouse seminal vesicle weights. The use of castrated host mice ensured that the testosterone production from the prepubertal human testis xenografts could be measured. 

**Figure 3:**
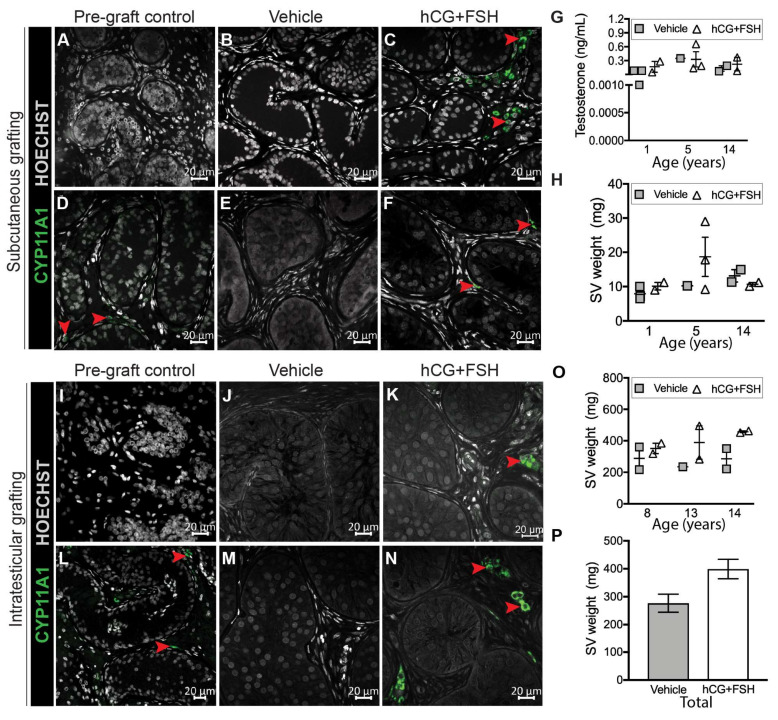
Steroidogenesis in prepubertal human testis xenografts. (**A**–**C**) Initiation of CYP11A1 expression in gonadotrophin-exposed subcutaneous xenografts (pre-graft control, 5-year-old). (**D**–**F**) Maintenance of CYP11A1 expression in gonadotrophin-exposed subcutaneous xenografts (pre-graft control, 1-year-old). (**G**) Plasma testosterone levels (*n* = 6-7 mice per group; *p* = 0.594) and (**H**) seminal vesicle (SV) weights (*n* = 6–7 mice per group; *p* = 0.431) in vehicle and gonadotrophin-exposed castrate host mice bearing subcutaneous xenografts. (**I**–**K**) Initiation of CYP11A1 expression in gonadotrophin-exposed intratesticular xenografts (pre-graft control, 8-year-old). (**L**–**N**) Maintenance of CYP11A1 expression in gonadotrophin-exposed intratesticular xenografts (pre-graft control, 14-year-old). (**O**) Seminal vesicle weight in vehicle and gonadotrophin-exposed intact host mice (*n* = 5–6 mice per group; *p* = 0.076) bearing intratesticular xenografts. (**P**) Graph shows overall mean data. Statistical significance was determined using two-way ANOVA. Data are presented as mean ± SEM (*n* = 3 prepubertal testis samples per transplantation site). Arrowheads indicate CYP11A1^+^ cells.

For subcutaneous xenografts, exposure to gonadotrophins (hCG + FSH) resulted in initiation (Figure 3a–c) and maintenance (Figure 3d–f) of CYP11A1 expression in a small proportion of interstitial cells. No CYP11A1 immunostaining was evident in any of the vehicle-exposed xenografts (Figure 3b,e), whereas CYP11A1^+^ cells were identified in gonadotrophin-exposed xenografts (Figure 3c,f). We further evaluated the endocrine activity of grafts by analysing testosterone levels and seminal vesicle weights in castrate recipient mice. There were no differences in plasma testosterone concentrations in mice that had been exposed to vehicle or gonadotrophins (0.13 vs. 0.25 ng/mL; *p* = 0.594; Figure 3g). The seminal vesicle weights were also similar between the vehicle- and gonadotrophin-exposed mice (10.02 vs. 13.93 mg; *p* = 0.431; Figure 3h). 

We also determined the effect of exogenous gonadotrophins on steroidogenesis in prepubertal human testis tissue that had been transplanted intratesticularly. Similar to subcutaneous xenografts, initiation (Figure 3i–k) and maintenance (Figure 3l–n) of CYP11A1 expression were observed in the interstitial space of intratesticular grafts exposed to gonadotrophins (Figure 3k,n), but not in vehicle controls (Figure 3j,m). As expected, seminal vesicles were in the normal adult range in mice grafted intratesticularly, reflecting intact endogenous testosterone production, and no difference was observed between vehicle- and gonadotrophin-exposed mice (277 vs. 399 mg; *p* = 0.076; Figure 3o,p). The prepubertal human testicular interstitium is mainly composed of round immature Leydig cells and spindle-shaped cells (fibroblast-like cells) [10]. We observed that upon gonadotrophin exposure, CYP11A1 was mainly restricted to clusters of round interstitial cells, whereas the majority of spindle-shaped interstitial cells appeared to be immunonegative for CYP11A1 (Figure S2).

Taken together, these results show that exposure to either exogenous or endogenous (mouse) gonadotrophins induces limited steroidogenic activity in prepubertal human testis grafts.

### 2.4. Effect of Exogenous Gonadotrophins on Sertoli Cell Maturation

The transition from immature to mature Sertoli cell is a stepwise process that involves (i) changes in the nuclear and cytoplasmic morphology, (ii) changes in protein expression (e.g., downregulation of AMH, upregulation of AR), (iii) a progressive decrease in the proliferative activity, and (iv) the establishment of the blood–testis barrier (BTB).

To determine the differentiation status of Sertoli cells in grafts, we investigated the immunoexpression profile of five markers [29,30,31].

#### 2.4.1. Expression of AR and AMH in Prepubertal Human Testis Grafts

**Figure 4:**
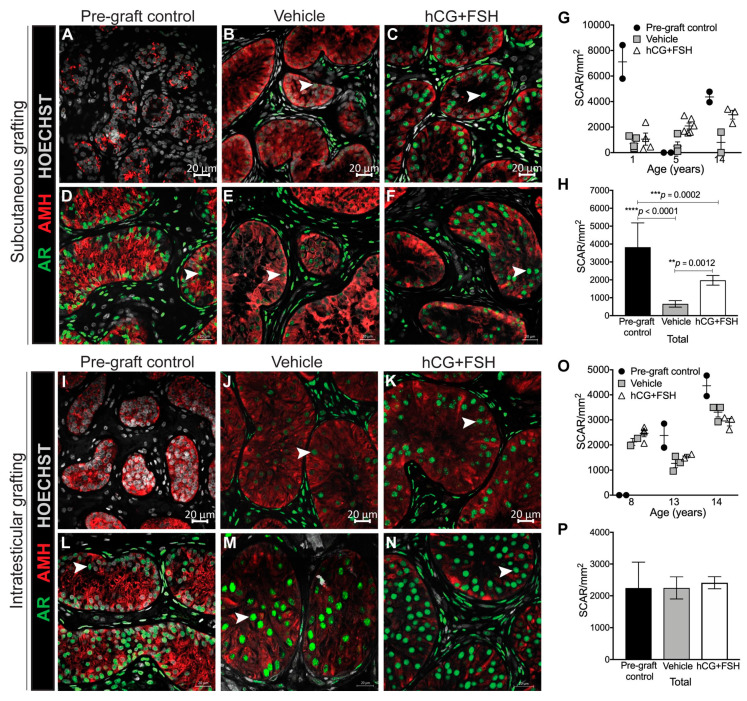
Immunoexpression of AR and AMH in prepubertal human testis xenografts. (**A**–**C**) Initiation of AR expression in gonadotrophin-exposed subcutaneous xenografts (pre-graft control, 5-year-old). (**D**–**F**) Maintenance of AR expression in gonadotrophin-exposed subcutaneous xenografts (pre-graft control, 1-year-old). (**G**) Individual data points represent the number of Sertoli Cell Androgen Receptor (SCAR) cells for each subcutaneous graft. (**H**) Graph shows overall mean data. (**I**–**K**) Initiation of AR expression in gonadotrophin-exposed intratesticular xenografts (pre-graft control, 8-year-old). (**L**–**N**) Maintenance of AR expression in gonadotrophin-exposed intratesticular xenografts (pre-graft control, 14-year-old). (**O**) Individual data points represent the number of SCAR for each intratesticular graft. (**P**) Graph shows overall mean data. Statistical significance was determined using two-way ANOVA. Data are presented as mean ± SEM (*n* = 3 prepubertal testis samples per transplantation site). Arrowheads point to AR^+^ Sertoli cells.

We sought to determine the impact of gonadotrophins on AR expression in prepubertal testis grafts. Sertoli cells begin to express AR as they progress from an undifferentiated to a more differentiated status [32]. Regardless of the transplantation site and treatment received, AR expression in Sertoli cells was induced in testis tissues obtained from two donors (5 and 8 years; Figure 4a–c,i–k), whilst its expression was maintained in transplanted testis tissues from the remaining patients across all experimental groups (Figure 4d–f,l–n). For subcutaneous xenografts, there was a significant reduction in the number of AR-expressing Sertoli cells between the pre-graft controls and subcutaneous xenografts (Figure 4g,h). However, the number of AR^+^ Sertoli cells in subcutaneous xenografts was lower in vehicle- compared with gonadotrophin-exposed xenografts (658 vs. 1975 cells/mm^2^; *p* = 0.0012; Figure 4g,h). Relative to pre-graft controls, the administration of exogenous gonadotrophins did not affect the number of AR-expressing Sertoli cells in intratesticular grafts. Similarly, there was no difference between the number of AR^+^ Sertoli cells in vehicle- and gonadotrophin-exposed intratesticular grafts (2247 vs. 2411 cells/mm^2^; *p* = 0.589; Figure 4o,p).

In the human testis, AMH expression declines at the onset of puberty [33]. A reduction in the intensity of AMH staining was observed in intratesticular grafts (Figure 4m,n); however, the remaining grafts maintained AMH expression regardless of the transplantation site or treatment received (Figure 4a–f,i–k). These data indicate that neither exogenous gonadotrophins (in castrated mice) nor endogenous testosterone (in intact host mice) are sufficient to induce complete AMH repression in prepubertal human testicular xenografts.

#### 2.4.2. Expression of SOX9 and Ki67 in Prepubertal Human Testis Grafts

**Figure 5:**
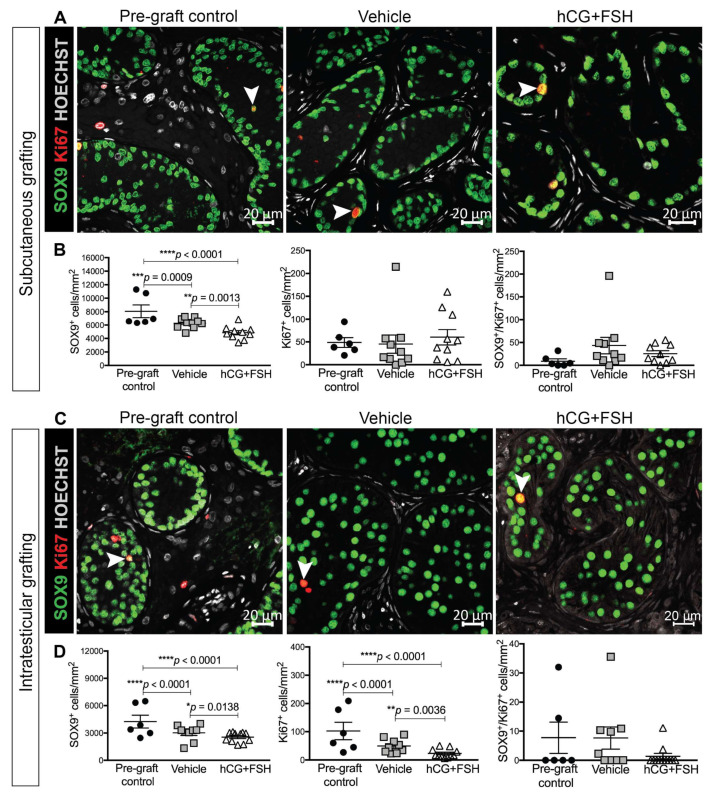
Proliferation of Sertoli cells in prepubertal human testis grafts. (**A**) Immunofluorescence for Sertoli cells (SOX9^+^) and proliferating cells (Ki67^+^) in subcutaneous xenografts. Pre-graft control (14 year old). (**B**) Quantification of proliferating Sertoli cells per square millimetre. (**C**) Immunofluorescence for Sertoli cells (SOX9^+^) and proliferating cells (Ki67^+^) in intratesticular xenografts. Pre-graft control (14 year old). (**D**) Quantification of proliferating Sertoli cells per square millimetre. Statistical significance was determined using two-way ANOVA. Data are presented as mean ± SEM (*n* = 3 prepubertal testis samples per transplantation site). Arrowheads point to SOX9^+^/Ki67^+^ Sertoli cells.

The Sertoli cell number in adulthood determines the capacity of the testis to support spermatogenesis [29]. Therefore, we determined the effect of gonadotrophins on Sertoli cell number in testis xenografts. SOX9 is present in Sertoli cells throughout all developmental ages. The number of SOX9^+^ cells per tubular area was significantly reduced in vehicle- and gonadotrophin-exposed xenografts compared with the equivalent pre-graft control for both subcutaneous and intratesticular xenografts (Figure 5a–d). Furthermore, the Sertoli cell numbers were significantly higher in vehicle-exposed xenografts compared with gonadotrophin-exposed xenografts for the subcutaneous (6303 vs. 4847 cells/mm^2^; *p* = 0.0013; Figure 5b) and intratesticular groups (3016 vs. 2549 cells/mm^2^; *p* = 0.0138; Figure 5d). 

The cessation of proliferative activity is an indicator of Sertoli cell maturation and is observed around puberty [31]. The total number of proliferating cells (Ki67^+^) was not significantly different between the pre-graft controls and subcutaneous xenografts exposed to either vehicle or gonadotrophins (Figure 5b). In addition, there was no difference in the number of Ki67^+^ cells in vehicle- compared with gonadotrophin-exposed controls (45.05 vs. 60.22 cells/mm^2^; *p* = 0.765; Figure 5b). However, for intratesticular xenografts there was a significantly lower number of proliferating cells (Ki67^+^) in both treatment groups compared to pre-graft controls (Figure 5d). Moreover, there was a higher number of Ki67^+^ cells in vehicle- compared with gonadotrophin-exposed controls (49.19 vs. 23.02 cells/mm^2^; *p* = 0.0036; Figure 5d).

To determine whether decreased Sertoli cell proliferation had caused the observed reduction in the Sertoli cell number, the co-expression of SOX9 and Ki67 was assessed in xenografts. Despite a reduction in the Sertoli cell number in xenografts, there was no significant difference in the SOX9^+^/Ki67^+^ cell number between pre-graft controls and treatment groups (Figure 5b,d). Similarly, there was no significant change in the SOX9^+^/Ki67^+^ cell number in vehicle- compared with gonadotrophin-exposed subcutaneous (43.41 vs. 25.61 cells/mm^2^, *p* = 0.518; Figure 5b) or intratesticular xenografts (7.65 vs. 1.35 cells/mm^2^, *p* = 0.112; Figure 5d). Overall, these data indicate that exposure to gonadotrophins reduced the Sertoli cell number, which may be due to increased apoptosis or a reduction in proliferation during an earlier period of gonadotrophin exposure.

#### 2.4.3. Expression of CX43 and CLDN11 in Prepubertal Human Testis Grafts

**Figure 6:**
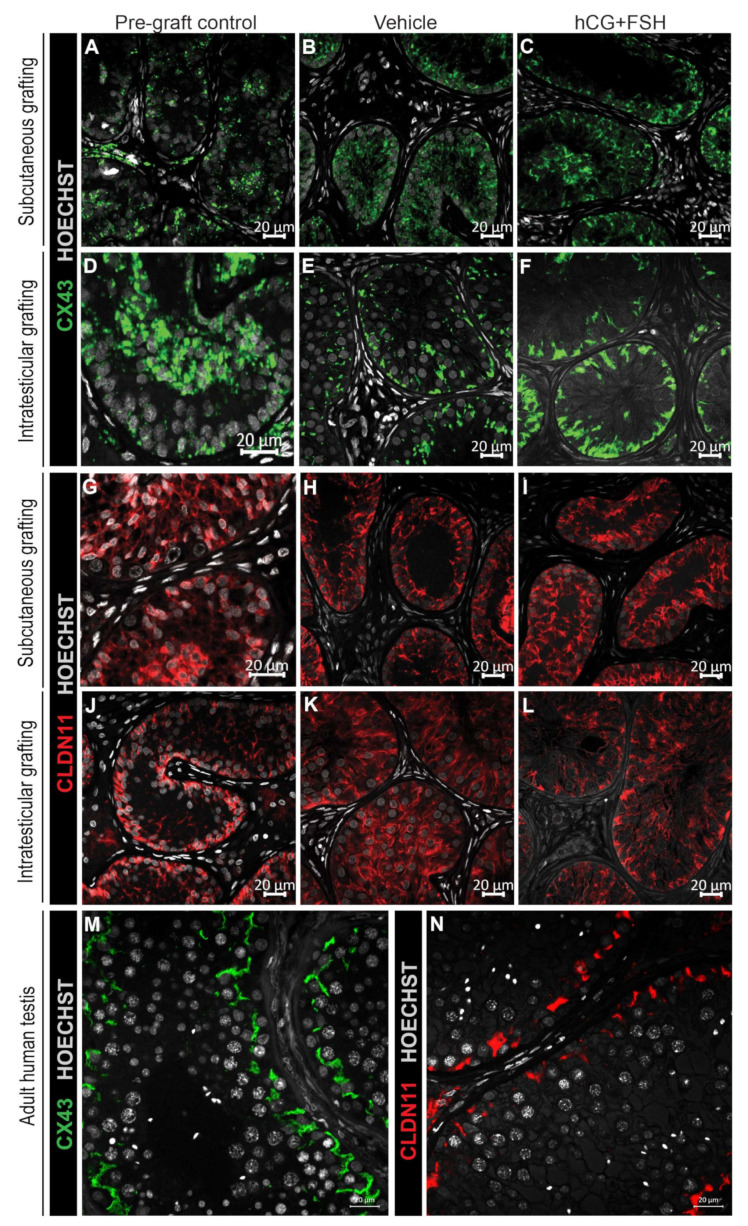
Expression of CX43 and CLDN11 in prepubertal human testis grafts. (**A**–**C**) Immature CX43 expression profile in subcutaneous grafts (pre-graft control; 1-year-old). (**D**–**F**) Partially mature CX43 expression profile in intratesticular grafts (pre-graft control; 14-year-old). (**G**–**I**) Immature CLDN11 expression pattern in subcutaneous grafts (pre-graft control; 1-year-old). (**J**) Partially mature CLDN11 expression pattern (pre-graft control; 14-year-old). (**K**,**L**) Immature CLDN11 expression pattern in intratesticular grafts. (**M**,**N**) Expression of CX43 and CLDN11 in adult human testis tissue.

Puberty marks the point at which the BTB is established [31]. An important feature of Sertoli cell maturation is the development of inter-Sertoli cell junctional complexes, which contribute to the establishment of the BTB. CX43 (gap junction protein) and CLDN11 (tight junction protein) exhibit distinct profiles of expression across different stages of testicular development. Diffuse CX43 and CLDN11 distribution is typical of immature seminiferous cords (prepubertal testis) prior to the formation of the BTB. As the BTB develops during puberty, both CX43 and CLDN11 become localised to the basal compartment of the seminiferous epithelium, and this is maintained into adulthood with associated spermatogenesis. For CX43 and CLDN11, the testis grafts displayed immature/partially mature staining patterns (Figure 6a–l), indicating that exogenous gonadotrophins did not induce the localised expression adjacent to the basement membrane observed in adult human testes (Figure 6m,n). 

### 2.5. Effect of Exogenous Gonadotrophins on Germ Cell Survival in Prepubertal Human Testis Grafts

**Figure 7:**
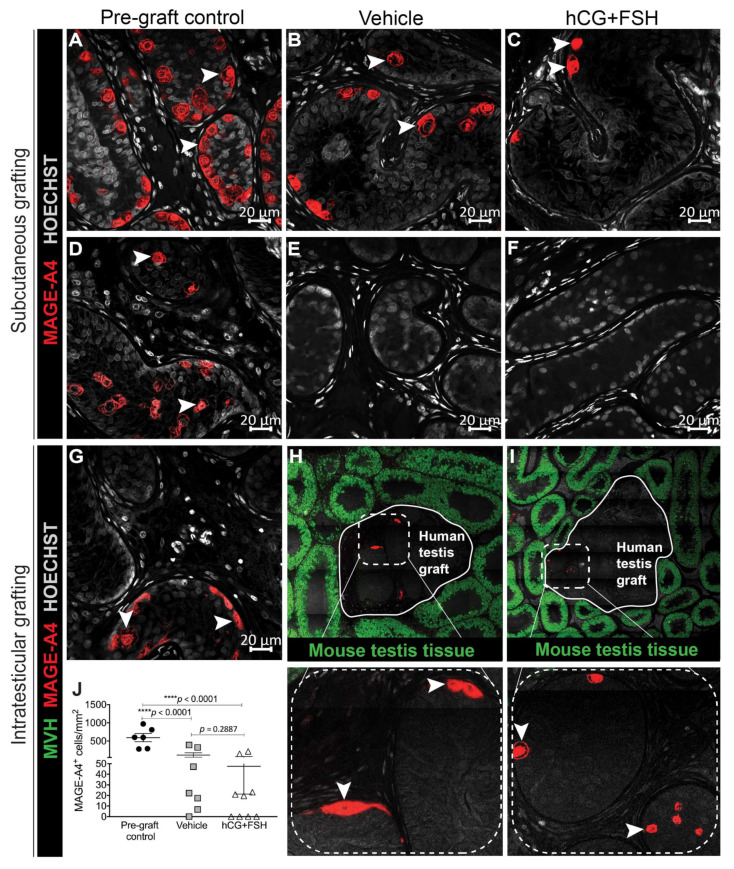
Expression of MAGE-A4 in prepubertal human testis grafts. (**A**) Pre-graft control (1-year-old) and subcutaneous xenografts exposed to (**B**) vehicle or (**C**) gonadotrophins. (**D**) Pre-graft control (14-year-old) and subcutaneous xenografts exposed to (**E**) vehicle or (**F**) gonadotrophins. (**G**) Pre-graft control (14-year-old) and intratesticular xenografts exposed to (**H**) vehicle or (**I**) gonadotrophins with corresponding higher magnification images (bottom images). (**J**) Quantification of human spermatogonia determined as the number of MAGE-A4^+^ cells per square millimetre. Individual data points represent the number of MAGE-A4^+^ cells for each pre-graft control and intratesticular graft. Statistical significance was determined using two-way ANOVA. Data are presented as mean ± SEM (*n* = 3 prepubertal testis samples per transplantation site). Mouse Vasa Homologue (MVH) is a germ cell-specific DEAD-box type RNA binding protein. MVH^+^ staining identifies mouse germ cells. Arrowheads indicate MAGE-A4^+^ cells.

At the time of xenotransplantation, spermatogonia were the most advanced germ cell type in the testes of all prepubertal patients. Grafts retrieved from the subcutaneous transplantation site showed variable spermatogonial survival. Melanoma-associated antigen 4 (MAGE-A4^+^) germ cells survived when the testis tissue from the 1-year-old patient was transplanted subcutaneously and exposed to vehicle or gonadotrophins (Figure 7a–c), whilst subcutaneous testis xenografts from the 5 and 14-year-old boys had no MAGE-A4^+^ germ cells within the seminiferous cords (Figure 7d–f).

When testis tissue was transplanted intratesticularly, spermatogonia were present in pre-graft controls (Figure 7g) and grafts (Figure 7h,i). However, there was no significant difference in the number of MAGE-A4^+^ cells in vehicle- compared to gonadotrophin-exposed xenografts (115.5 vs. 47.5 cells/mm^2^; *p* = 0.2887; Figure 7j). 

## 3. Discussion

Development of immature germ cells into sperm is a dynamic process that occurs at puberty and is dependent on the (i) ability of adult Leydig cells to produce testosterone [11,14,34,35] and (ii) establishment of a fully mature Sertoli cell population [14,31,36,37,38,39]. One of the main objectives of the present study was to evaluate the effect of a pubertal induction regimen with exogenous gonadotrophins on the steroidogenic activity and Sertoli cell maturation in prepubertal human testis xenografts. Moreover, we aimed to determine whether the transplantation site has an impact on overall testicular xenograft development. 

To examine the steroidogenic potential of testicular interstitial cells, prepubertal human testis pieces were transplanted subcutaneously and exposed to exogenous gonadotrophins or treated with the corresponding vehicle. The castration of host mice was performed to remove any confounding influence of endogenous mouse testicular testosterone and to ensure that plasma testosterone and seminal vesicle measurement reflected the testosterone production by the subcutaneous grafts. Occasional steroidogenically active cells were seen in the interstitium of gonadotrophin-exposed grafts, but not in vehicle-exposed transplants. Furthermore, plasma testosterone levels and seminal vesicle weights remained relatively low in the castrate recipient mice. A previous study has demonstrated occasional 3β-hydroxysteroid dehydrogenase (3β-HSD, steroidogenic enzyme) staining when fresh or frozen-thawed prepubertal human testis tissues were grafted into the scrotum of untreated intact mice [17]. Limited steroidogenesis in these untreated xenografts may have resulted from a longer xenografting period [17].

The normal prepubertal human testicular interstitium is devoid of mature adult Leydig cells and is composed of immature Leydig cells and numerous fibroblast-like cells (adult Leydig cell precursors), with the latter accounting for the majority of the interstitial cells (91%) in the prepubertal human testis [10,40]. In our study, we hypothesise that CYP11A1-positive cells detected in the prepubertal testis xenografts are immature Leydig cells that have become steroidogenically active when exposed to exogenous gonadotrophins, rather than fibroblast-like cells that have differentiated into an “adult” Leydig cell population. This is in keeping with early ultrastructural studies, which showed that, in the prepubertal human testicular interstitium, only immature Leydig cells exhibit the morphological features of steroid-producing cells [10]. It could, therefore, be speculated that in our study, the fibroblast-like precursors did not differentiate to fully mature testosterone-producing Leydig cells and that the immature Leydig cells were capable of producing low levels of testosterone. Interestingly, it has been shown in rodents that LH is unlikely to be the initial stimulus for the development of adult Leydig cell precursors, but once the differentiation has started adult Leydig cells require LH-stimulation [40,41,42,43]. This concept is further supported by the expression of a truncated, nonfunctional form of LHCGR in adult Leydig cell precursors [13,44,45]. In line with our findings, Rivarola and colleagues reported that the testicular cells isolated from 1-7-month-old infants under in vitro hLH stimulation increased their testosterone secretion, whereas the testicular cells from 12-36-month-old children did not respond to hLH [46]. The same authors suggested that this result could be attributed to the very few steroid-producing interstitial cells present in 12-36-month-old boys in comparison with testosterone-producing Leydig cells in the testes of young infants. Our previous study using human foetal testis xenografts supports this hypothesis; continuous gonadotrophin (hCG) stimulation for 9-12 months resulted in a significant increase in the seminal vesicle weight of host castrate mice compared with those in which hCG was withdrawn during the grafting period [32]. 

Interestingly, Chen and colleagues showed that development of precursors into testosterone-producing rat adult Leydig cells requires stimulation by paracrine factors from the seminiferous tubules [47]. Cell-specific ablation studies in mice suggest that Sertoli cells may be the source of key factors required for the differentiation of precursors into adult Leydig cells [48]. The role of Sertoli cell-derived AMH in postnatal life remains unclear, though studies in rodents suggest that AMH may serve as a negative modulator of Leydig cell differentiation and function [49,50]. The injection of AMH into adult Leydig cell-ablated rat testes (precursors are resistant to the ablation) resulted in the inhibition of the proliferation and differentiation of Leydig cell precursors [51]. Furthermore, the overexpression of AMH in male mice was shown to block adult Leydig cell differentiation [49], which may occur directly via a functional AMH receptor II abundantly expressed on precursor Leydig cells [52]. The limited steroidogenic activity in gonadotrophin-exposed grafts in our study may be in part due to the retention of AMH in Sertoli cells, which could act as a negative regulator of Leydig cell differentiation; however, this would require further investigation.

At puberty, an inverse correlation is observed between serum testosterone and AMH; as a consequence, the default assumption is that, in humans, testosterone represses AMH expression in Sertoli cells [53,54,55]. Nevertheless, direct evidence supporting the hypothesis that testosterone causes AMH inhibition in human testis is still required. To evaluate the effect of testosterone on AMH in AR-positive prepubertal testicular grafts, we took advantage of intact mice, which produce intratesticular testosterone. Seminal vesicle weights were within the normal range for intact adult mice [56,57], indicating that intratesticular xenografts were exposed to testosterone produced from host mice. Furthermore, AR expression was induced/maintained in xenografts indicating acquisition of androgen-sensitivity. In the present study, we showed clearly that AMH expression was detectable, despite the presence of endogenous testosterone and AR expression in prepubertal human testis tissue transplanted into intact mice. These results concur with those seen in a previous study showing AMH expression in AR-positive prepubertal testis grafts [19]. Maintenance of AMH expression in AR-expressing seminiferous cords has also been documented in studies involving long-term human foetal testis xenotransplants, in which host mice were exposed to exogenous hCG [32]. Moreover, it seems that no androgen response elements are present on the human AMH promoter [58], and studies on Sertoli-cell specific AR knockout and gain-of-function transgenic mouse models demonstrated that androgen action is not required for AMH downregulation [37,59]. Taken together, these data indicate that androgen-independent mechanisms may contribute to the inhibition of AMH in human Sertoli cells. 

Exposure of prepubertal testis grafts to gonadotrophins did not repress the expression of markers (AMH, Ki67) found in immature Sertoli cells or induce the formation of the BTB. In our previous work, Sertoli cells displayed a mature CX43 expression pattern in human foetal testis grafts exposed to hCG for 9-12 months [32]. Although the exact mechanism underpinning CX43 expression is not entirely clear, data reported in our previous study indicate that testosterone produced from human foetal testis xenografts may have stimulated the initial expression and localisation of CX43 restricted to the basement membrane, similar to that observed in adult testes. On the other hand, human foetal testis grafts that had hCG withdrawn for the final 5 months showed limited steroidogenic activity and faint CX43 staining. Together, these findings suggest that the lack of expression of a mature CX43 profile in prepubertal human testis grafts exposed to gonadotrophins could potentially be due to low testosterone levels produced from grafts or insufficient mouse intratesticular testosterone. Neither human foetal testis grafts exposed to hCG nor prepubertal testis grafts exposed to combined gonadotrophins (hCG and FSH) displayed a mature CLDN11 expression profile, suggesting that the immature human testis tissue may require additional factors to induce the fine linear CLDN11 staining at the basal compartment, indicative of Sertoli cell maturation. Longer periods of grafting or the use of an alternative hormonal regimen may potentially induce maturational changes in CLDN11 expression

Xenografted testis tissue exhibited a reduction in the Sertoli cell and spermatogonial numbers compared to pre-graft controls. This may be partially explained by an initial loss due to ischemia and hypoxia that occurs during the first days after transplantation [60]. Poor overall survival of peripubertal testis tissue with ongoing meiosis in the pre-graft material could be attributed to increased sensitivity to ischemia and decreased angiogenic activity, indicating that the developmental stage of the testis at the time of grafting affects the outcome of testis tissue xenotransplantation [17,61,62,63]. In the present study, incomplete Sertoli and Leydig cell maturation might provide an explanation for the lack of germ cell differentiation in prepubertal human testis transplants. It is possible that grafts require a longer post-transplantation period; indeed, previous long-term studies have reported initiation of meiosis in testis grafts [17,18,19,64]. Overall, the site of transplantation did not appear to affect the Sertoli cell maturation and the responsiveness of interstitial cells to exogenous gonadotrophins. When comparing the xenotransplantation sites, the most favourable location for germ cell survival appeared to be the mouse testicular parenchyma. Lower testicular temperature compared to the dorsal skin, testosterone, and/or other local factors released from the testis of intact hosts might have supported germ cell survival in testis fragments transplanted intratesticularly; however, further studies are required to confirm this result.

## 4. Materials and Methods

### 4.1. Ethics Statement

Ethical approval for this study was granted by the South East Scotland Research Ethics Committee (LREC13/SS/0145) and the internal review board of the UZ Brussel (B.U.N.143201422558). Written informed consent was given for the use of human testis tissue for research. 

### 4.2. Animals

For all studies, animals (*n* = 37; aged 4–6 weeks, CD1 and Swiss Nu/Nu mice, Charles River UK, Charles River Belgium) were maintained under standard conditions of care and use with access to food and water ad libitum. Studies were performed according to the Animal (Scientific Procedures) Act 1986 under UK Home Office project licence approval (P5B09956A) and the Animal Care and Use Committee of the Vrije Universiteit Brussel (14-216-4).

### 4.3. Subcutaneous Grafting Procedure

Fresh and frozen-thawed testis tissue fragments (3–6 pieces per host mouse) from six patients aged 1–14 years were grafted subcutaneously into male nude host mice (*n* = 33). During the castration and transplantation, mice were placed on a heating plate at 37 °C to avoid anaesthesia-induced hypothermia. Mice were anaesthetised by the inhalation of isoflurane or intraperitoneal injection (75 μL/10 g of body weight) of medetomidine hydrochloride (0.1 mg/mL; Virbac Animal Health, Waver, Belgium) and ketamine hydrochloride (0.75 mg/mL; Ceva Santé Animale, Brussels, Belgium). Castration (*n* = 21 mice) was performed the same day as xenografting. The surgical area was cleaned with 70% ethanol. A longitudinal scrotal incision was made to remove the testicular fat pad, testes were exteriorised, and the spermatic cords were tied off using Mersilk 3.0 silk suture (Ethicon, Livingston, UK). The skin was closed using 3.0 sutures. Testis fragments (~1 mm^3^) were inserted subcutaneously under the dorsal skin of the host nude mouse using a 13G cancer implant needle (Popper and Sons, New York, NY, USA). Mice were kept on the heated surgical table until they fully recovered from the effect of anaesthesia. Subsequently, the mice were housed in individually ventilated cages. After surgery, the mice received either an analgesic (Rimadyl SA, Pfizer, New York, NY, USA; 0.5 mL/250 mL) or antibiotic (Baytril, Enrofloxacin; Bayer, Leverkusen, Germany; 1 mL/250 mL) in their drinking water for 5 days or the subcutaneous injections of antibiotics (Baytril 2.5% diluted 1:10, Bayer, Brussels, Belgium) and analgesics (2 mg/kg; Metacam, Boehringer Ingelheim, Brussels, Belgium) for 3 days after surgery. 

### 4.4. Intratesticular Grafting Procedure

The same pre-/post-surgery steps and recovery procedures used for subcutaneous grafting were applied to intratesticular xenotransplantation. Fresh and frozen-thawed testis tissues (1–2 pieces per intact mouse) from four patients aged 8–14 years were transplanted intratesticularly into intact host mice (*n* = 16). Following anaesthesia, the surgical area was cleaned with 70% ethanol. A small mid-abdominal incision was made using sterile scissors. Sterile forceps were used to grasp the skin, the testes were then exteriorised, and a small incision in the tunica albuginea was made to allow the insertion of one pre(peri)pubertal human testis fragment (~1 mm^3^) per mouse testis. The tunica was closed using non-absorbable 8.0/10.0 sutures (Ethicon, Livingston, UK; Ethicon, Instruvet, Beringen-Paal, Belgium). The skin was sutured closed using 3.0 sutures (Ethicon, Livingston, UK). 

### 4.5. Treatment of Host Mice

To mimic the pubertal hormonal environment, mice grafted with pre(peri)pubertal human testicular pieces commenced one week after the grafting, subcutaneous injections of hCG (0.1 mL/20 IU; Ovitrelle, Merck Serono, Feltham, UK) and FSH (0.1 mL/12.5 IU; Bemfola, Gedeon Richter, London, UK) or vehicle (0.1 mL water for injections; Henry Schein Medical, Gillingham, UK). Injections were continued three times a week for 12 weeks. In total, 3 mice (8%) died before the experiments were completed and 3 mice (8%) were culled early to alleviate potential suffering after showing early signs of ill health. An overview of the study design and treatment groups can be found in Table 2. 

### 4.6. Retrieval of Xenografts and Seminal Vesicles

Mice were culled after 13 weeks either by the inhalation of carbon dioxide (CO_2_) followed by cervical dislocation under schedule 1 of the Animal (Scientific Procedures) Act 1986 or by cervical dislocation under deep anaesthesia. 

In the case of intratesticular grafting, mouse testes were collected and fixed in Bouin’s solution (Clin-Tech Ltd, Guildford, UK) for 6 h. Grafts were dissected from the back skin of host mice, individually weighed, and placed in Bouin’s fixative for 2–4 h at room temperature. Seminal vesicle weights were recorded as an indicator of the bioactive testosterone produced by xenografts [56,61].

### 4.7. Measurement of Plasma Testosterone Levels

Blood was collected from host mice via cardiac puncture using syringes pre-coated with heparin and the plasma then separated by centrifugation. Testosterone (T) was extracted from mouse plasma by solid phase extraction LC-MS/MS using 10 mg of hydrophilic-lipophilic balanced (HLB) Oasis cartridges (10 mg, Waters, Wilmslow, UK). Mouse plasma (100 µL) was enriched with 1 ng of ^13^C_3_-Testosterone (^13^C_3_-T; CDN Isotopes) to track the analyte chromatographically and as an internal standard. A calibration curve using a 1% BSA surrogate matrix was prepared covering the range 0.002–10 ng/mL of T, also enriched with ^13^C_3_-T (1 ng). HLB cartridges were conditioned with methanol (1 mL) and water (1 mL) and the biological sample was loaded onto the cartridge, washed with water (1 mL) and 5% methanol (1 mL), and then eluted with methanol (1 mL). The eluate was reduced to dryness under nitrogen at 40 °C and reconstituted in water/methanol (45:55, v/v, 100 µL). The sample was analysed by LC-MS/MS. Chromatographic separation was achieved by injecting 20 µL of the extract onto a Shimadzu Nexera UPLC system fitted with an ACE Excel C18-PFP column (150 × 2.1 mm; 2 µm, ACT Technologies, Aberdeen, UK) protected by a Kinetex KrudKatcher (Phenomenex, Macclesfield, Cheshire, UK) and maintained at 30 °C. The mobile phase consisted of 0.1% formic acid (Sigma Aldrich, Gillingham, UK) in water (A) (LC-MS grade, Fisher Scientific, Loughborough, UK) and 0.1% formic acid in methanol (B) (LC-MS grade, Fisher Scientific, Loughborough, UK) at a flow rate of 0.5 mL/min. Gradient elution was from 45% to 80% B, with a total run time of 14 min. Testosterone and ^13^C_3_-T were eluted at 5.7 min while ensuring the temporal separation of the isomers epi-testosterone (4.8 min) and dehydroepiandrosterone (6.07 min) and isotopologues of androstenedione (5.1 min). Following separation, mass analysis was carried out on a QTrap 6500+ linear ion trap tandem mass spectrometer (AB Sciex, Warrington, UK) operated in positive ion electrospray mode (5.5 kV, 550 °C, ion source gas 1 and 2; 60 and 40). T specific transitions monitored were m/z 289.1 → 97.0, 109.2 and ^13^C_3_-T transitions were *m/z* 292.1 → 100.2, for quantitative and qualitative ions. Chromatographic peaks were integrated using Analyst 1.6 Software. Linear regression analysis was applied to the ratio of the peak area of T/^13^C_3_-T in the calibration curve and the biological samples using Analyst 1.6 Quantitation software. The method was validated according to the European Medicines Agency bioanalytical method validation guidelines for accuracy, precision, sensitivity, selectivity, parallelism, range, reproducibility, and stability. 

### 4.8. Histology and Immunostaining

For histological analyses, testis sections were stained with Haematoxylin and Eosin (H&E) following standard protocols [32]. Specific proteins were detected using immunohistochemical methods, as previously described [32,65]. Negative controls were included in each experiment, for which the primary antibodies were omitted and replaced with the appropriate blocking serum, and in all such cases the immunostaining was negative. The primary antibodies used were: AMH (MIS C-20) (1:1000; Santa Cruz Biotechnology; sc-6886; Heidelberg, Germany); AR (N-20) (1:2000; Santa Cruz Biotechnology; sc-816; Heidelberg, Germany); SOX9 (1:10.000; Millipore; AB5535; Watford, UK); Connexin 43 (1:300; Cell Signaling Technology; 3512; London, UK); Claudin 11 (1:500; Thermo Fisher Scientific; 36-4500; Loughborough, UK); CYP11A1 (1:5000; Sigma-Aldrich; HPA016436; Gillingham, UK); MVH/DDX4 (1:300; Abcam; ab13840; Cambridge, UK); MAGE-A4 (1:200; gift from Professor Giulio Spagnoli, University of Basel); Ki67 (1:100; Abcam; ab16667; Cambridge, UK); Vimentin (1:100; Dako; M072501; Heverlee, Belgium). Secondary antibodies used were: chicken anti-mouse IgG-HRP (1:200; Santa Cruz Biotechnology; sc-2954); chicken anti-rabbit IgG-HRP (1:200; Santa Cruz Biotechnology; sc-2862); chicken anti-goat IgG-HRP (1:200; Santa Cruz Biotechnology; sc-2953). Visualisation was performed using Tyramide Signal Amplification (1:50; PerkinElmer; TSA Plus Fluorescein, TSA Plus Cyanine 3, TSA Plus Cyanine 5) according to the manufacturer’s instructions. Slides were counterstained with Hoechst (1:4000; Thermo Fisher Scientific; 33342; Loughborough, UK) and mounted with PermaFluor (Thermo Fisher Scientific; Loughborough, UK) prior to imaging. 

### 4.9. Image Acquisition and Analysis

Fluorescent images were captured using an LSM780 confocal microscope (Carl Zeiss, Cambridge, UK) with Zen imaging software (Carl Zeiss, Cambridge, UK), whereas nonfluorescent images were acquired using a Provis AX70 microscope (Olympus Optical, London, UK) fitted with a Cannon DS6031 digital camera. The total number of positively stained cells was quantified and expressed relative to the total section area when a specific marker was expressed in both compartments (interstitium and seminiferous cords), or relative to the total tubular area when the protein of interest was located within the seminiferous cords. 

### 4.10. Statistical Analysis

Data were analysed using GraphPad Prism (San Diego, CA, USA). A two-way ANOVA test was performed to account for two independent variables, the inter-individual variation between the prepubertal testis samples and also the variation between the testis grafts, as described previously [66,67]. The criterion for significance was *p* < 0.05.

## 5. Conclusions

In summary, we have shown limited Leydig cell steroidogenic function and Sertoli cell maturation in prepubertal human testicular xenografts following a pubertal induction regimen of gonadotrophin stimulation. Furthermore, despite AR being present in Sertoli cells, endogenous testosterone did not repress AMH expression in xenografts. Proliferating cells were identified in pre-graft controls and grafts, consistent with clinical studies indicating that the prepubertal human testis is not quiescent but it is susceptible to damage by chemotherapy and radiotherapy. Despite exposure to a gonadotrophin regimen aimed at mimicking human puberty, germ cell differentiation and spermatogenesis were not achieved in xenografts. These data provide a basis for future studies aimed at identifying the optimal hormonal environment and additional factors that will support the functional maturation of the SSC niche. Identification of these factors is critical for the development of transplantation as a model for fertility preservation in male survivors of childhood cancer.

## Figures and Tables

**Table 1:** Patient characteristics.

Age (yrs)	Diagnosis	Pre-Biopsy Chemotherapy	Biopsy Condition	Most Advanced Germ Cell Type
1	Ependymoma	No	Fresh	Spermatogonia
5	Medulloblastoma	No	Fresh	Spermatogonia
8	Aplastic Anaemia	No	Cryopreserved	Spermatogonia
13	Myelodysplastic Syndrome	No	Cryopreserved	Spermatogonia
13	Acute Lymphoblastic Leukaemia	Yes *	Cryopreserved	Spermatocytes
14	Anaplastic Large Cell Lymphoma	Yes **	Fresh	Spermatogonia

* Methotrexate; ** Vinblastine, Bleomycin, Methotrexate, Adriamycin.

**Table 2:** Xenografting schedule and graft retrieval rates.

	Subcutaneous - Castrate		Subcutaneous - Intact		Intratesticular - Intact	
**Treatment**	Vehicle	hCG+FSH	Vehicle	hCG+FSH	Vehicle	hCG+FSH
**Testis Tissue**	*n* = 6	*n* = 6	*n* = 3	*n* = 3	*n* = 4	*n* = 4
**Patient Age (Years)**	1, 5, 8, 13, 13, 14	1, 5, 8, 13, 13, 14	8, 13, 13	8, 13, 13	8, 13, 13, 14	8, 13, 13, 14
**Recipient Mouse**	*n* = 10	*n* = 11	*n* = 6	*n* = 6	*n* = 8	*n* = 8
**Graft Recovery Rate (%)**	15/43 (35)	26/47 (55)	4/18 (22)	4/18 (22)	9/16 (56)	11/15 (73)

Fresh testis tissue: 1, 5, 14 years; frozen-thawed testis tissue: 8, 13, 13 years.

## Supplementary Materials

The following are available online at https://www.mdpi.com/2072-6694/12/10/2830/s1: Figure S1: Expression of MAGE-A4 in peripubertal human testis tissue (13-year-old). Figure S2: Expression of CYP11A1 in prepubertal human testis grafts.
