# Supplementary material for: Fertility Preservation in Childhood Cancer: Endocrine Activity in Prepubertal Human Testis Xenografts Exposed to a Pubertal Hormone Environment
Source: Cancers (Basel). 2020 Sep 30;12(10):2830. doi: 10.3390/cancers12102830 (PMC7600569; doi:10.3390/cancers12102830)
Supplement: Supplementary file 1 [file cancers-12-02830-s001.pdf]

cancers

Supplementary Figures

Fertility Preservation in Childhood Cancer: Endocrine Activity in Prepubertal Human Testis Xenografts Exposed to a Pubertal Hormone Environment

Marsida Hutka, Prashant Kadam, Dorien Van Saen, Natalie Z. Homer, Jaime Onofre, W. Hamish B. Wallace, Lee B. Smith, Jan-Bernd Stukenborg, Ellen Goossens and Rod T. Mitchell

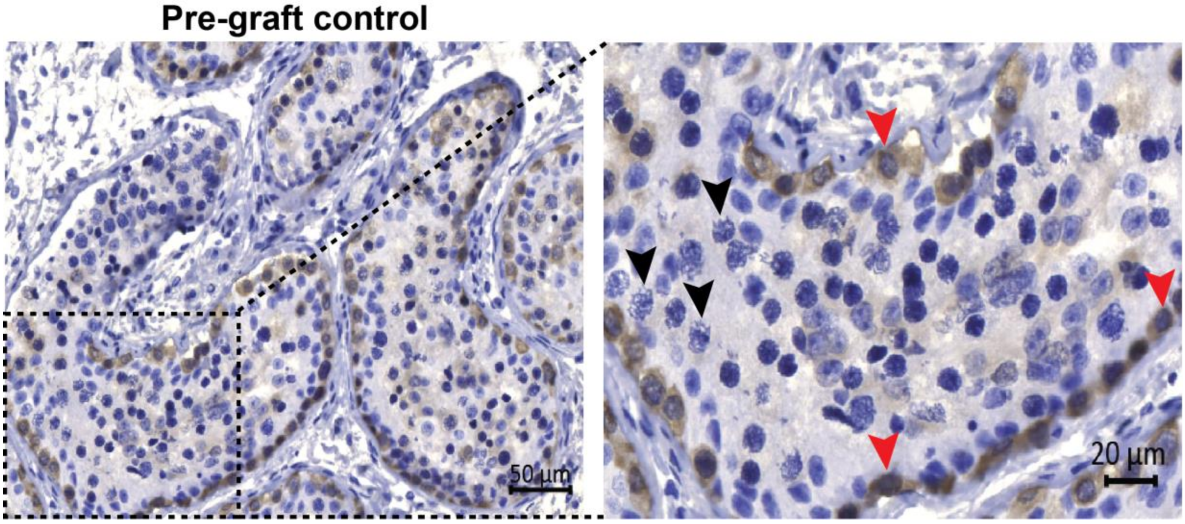

Figure S1. Expression of MAGE-A4 in peripubertal human testis tissue (13-year-old). Higher magnification image on the right shows the presence of spermatogonia (MAGE-A4⁺) and spermatocytes within the seminiferous tubule. Red arrowheads point to spermatogonia. Black arrowheads point to spermatocytes. Scale bars: 20 µm and 50 µm.

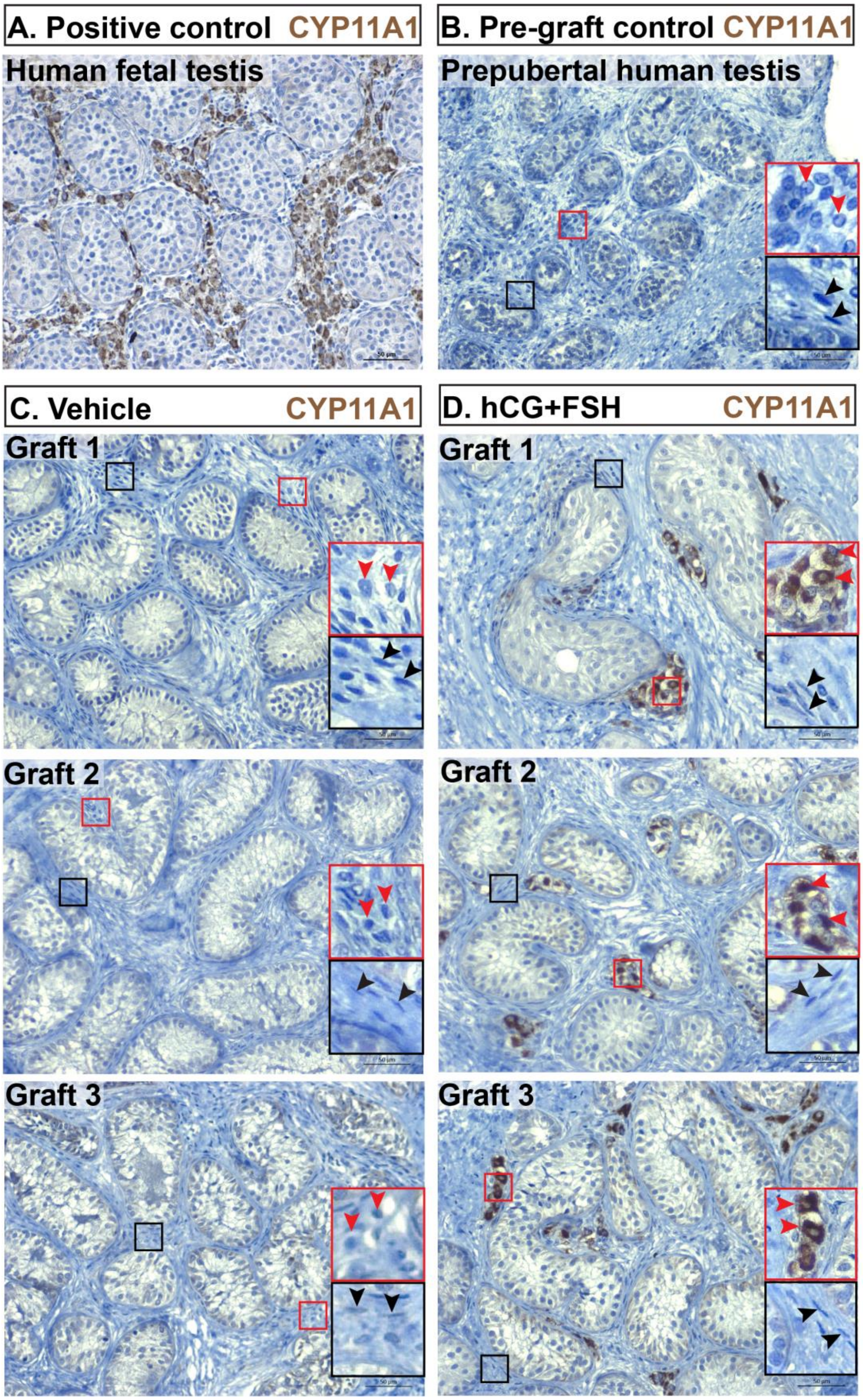

Figure S2. Expression of CYP11A1 in prepubertal human testis grafts. (**A**) Positive control (second-trimester human fetal testis). (**B**) Pre-graft control (5-year-old). (**C**) Subcutaneous grafts exposed to vehicle for 12 weeks. (**D**) Subcutaneous grafts exposed to gonadotrophins for 12 weeks. Red arrowheads point to round-shaped interstitial cells. Black arrowheads point to spindle-shaped interstitial cells. Scale bars: 50 µm.
